# A review of genetic variant databases and machine learning tools for predicting the pathogenicity of breast cancer

**DOI:** 10.1093/bib/bbad479

**Published:** 2023-12-26

**Authors:** Rahaf M Ahmad, Bassam R Ali, Fatma Al-Jasmi, Richard O Sinnott, Noura Al Dhaheri, Mohd Saberi Mohamad

**Affiliations:** Health Data Science Lab, Department of Genetics and Genomics, College of Medical and Health Sciences, United Arab Emirates University, Tawam road, Al Maqam district, Al Ain, Abu Dhabi, United Arab Emirates; Health Data Science Lab, Department of Genetics and Genomics, College of Medical and Health Sciences, United Arab Emirates University, Tawam road, Al Maqam district, Al Ain, Abu Dhabi, United Arab Emirates; Health Data Science Lab, Department of Genetics and Genomics, College of Medical and Health Sciences, United Arab Emirates University, Tawam road, Al Maqam district, Al Ain, Abu Dhabi, United Arab Emirates; Division of Metabolic Genetics, Department of Pediatrics, Tawam Hospital, Al Ain, United Arab Emirates; School of Computing and Information System, Faculty of Engineering and Information Technology, The University of Melbourne, Melbourne, Victoria, Australia; Health Data Science Lab, Department of Genetics and Genomics, College of Medical and Health Sciences, United Arab Emirates University, Tawam road, Al Maqam district, Al Ain, Abu Dhabi, United Arab Emirates; Division of Metabolic Genetics, Department of Pediatrics, Tawam Hospital, Al Ain, United Arab Emirates; Health Data Science Lab, Department of Genetics and Genomics, College of Medical and Health Sciences, United Arab Emirates University, Tawam road, Al Maqam district, Al Ain, Abu Dhabi, United Arab Emirates

**Keywords:** pathogenicity prediction, genetic variants database, machine learning, artificial intelligence, breast cancer, data science

## Abstract

Studies continue to uncover contributing risk factors for breast cancer (BC) development including genetic variants. Advances in machine learning and big data generated from genetic sequencing can now be used for predicting BC pathogenicity. However, it is unclear which tool developed for pathogenicity prediction is most suited for predicting the impact and pathogenicity of variant effects. A significant challenge is to determine the most suitable data source for each tool since different tools can yield different prediction results with different data inputs. To this end, this work reviews genetic variant databases and tools used specifically for the prediction of BC pathogenicity. We provide a description of existing genetic variants databases and, where appropriate, the diseases for which they have been established. Through example, we illustrate how they can be used for prediction of BC pathogenicity and discuss their associated advantages and disadvantages. We conclude that the tools that are specialized by training on multiple diverse datasets from different databases for the same disease have enhanced accuracy and specificity and are thereby more helpful to the clinicians in predicting and diagnosing BC as early as possible.

## INTRODUCTION

Machine learning (ML) is a subset of artificial intelligence that uses input data to learn patterns through many widely available algorithms and models. The challenges for analyzing and interpreting ever increasing volumes of data (big data) are increasing. Consequently, there is a need for novel ML tools to optimally process and learn from such big data. One emerging ML approach that is currently receiving much attention is deep learning (DL) [1]. It describes a family of algorithms/models, typically including multi-layer neural networks with many hidden units [2]. Such models can be used to learn complex patterns that can, for example, support predictions [2].

Advances in technology, have changed the understanding of the available sequenced human genetic variants. Since the first human genome was sequenced, many more have been sequenced in academic, clinical and the private sector settings [3]. The number of rare variants is also growing and there is a pressing need to determine whether variants are pathogenic or benign.

In this context, breast cancer (BC) is one of the most common tumor types in the world [4]. In women between 20 and 50 years old, BC represents around 11% of all cancer mortalities [5], while in men, it is 19% higher compared with women [6]. The early diagnosis of BC to reduce the mortality rate is essential. In the field of medical analysis, ML algorithms have been extensively applied [7] with examples in predicting coronavirus disease (COVID)-19 [8], Alzheimer’s progression [9], chronic diseases [9], liver disorders [10], heart disease [11], cancer [12] and others [13, 14]. The use of DL and ML for BC prediction is constantly advancing. The key factor in developing ML tools for BC lies in training them with specific BC data, rather than the algorithms themselves. Choosing the right ML tool for BC prediction is challenging due to the variability in datasets, which can impact the performance of ML models based on the training data [15]. A number of studies have explored ML prediction techniques for BC; however, these have not considered the pathogenicity of gene variants.

Human genetic variant databases serve as repositories of extensive data concerning thousands of human genetic variants, encompassing diverse information and purposes, from disease prediction [16] to supporting personalized medicine [17]. These databases, such as 1000 Genomes [18], COSMIC [19], ClinVar [20] and SwissVar [21] not only share variant-associated data but also maintain their unique annotations and datasets, resulting in heterogeneity across them. This diversity poses challenges in terms of data structure and consistency for geneticists, biologists and clinicians [22].

While previous efforts have integrated variant data from next-generation sequencing (NGS) for specific tools and workflow pipelines [23], focusing solely on sequence-related information has demonstrated limitations in accuracy [24]. Pathogenic variations can alter a protein’s structural features, particularly disulfide bond sites [25], and impact protein stability [26]. Understanding the effects of variants on protein stability is crucial, necessitating an exploration of a protein’s structure, function and dynamic relationships. Despite the success of 3D structure classifiers [27–29], sequence-based methods outpace structure-based modeling methods in assessing the effects of single amino acid variants (SAVs).

This review aims to highlight the genetic variant databases and associated ML tools used for prediction of BC pathogenicity. We first summarize well-known BC gene variants including their location and function and the associated abnormality of each genetic variant. Following this, a review of databases used in this type of research is explored including the targeted disease, the accessibility, their advantages and disadvantages and the associated website of each database. An example of applying the databases for predicting BC pathogenicity is provided and discussion of the advantages and disadvantages of each database provided. Moreover, we describe the ML tools, the advantages and disadvantages and the algorithms underpinning each tool along with the tool accessibility.

## GENETIC VARIANTS DATABASES

The American College for Medical Genetics (ACMG) and the Association for Molecular Pathology (AMP) have issued guidelines to classify the challenging missense variants or variants of unknown significance (VUS) as pathogenic or benign [30]. This is a consequence of the rarity of missense variants and hence the lack of data-driven clinical evidence, such as segregation and case control. As the problem of VUS has grown over time, most clinical genetic tests reported in ClinVar [20] are VUSs, even among highly studied cancer predisposition genes like Breast Cancer 1 (*BRCA1*), Tumor Protein 53 (*TP53*) and Phosphatase and Tensin Homolog (*PTEN*) [20].

**Figure 1:**
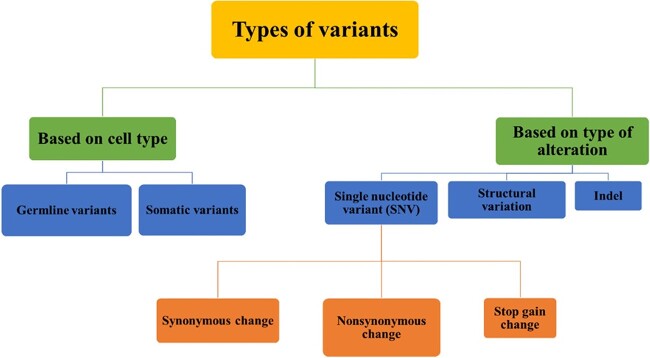
The types of variants based on cell type and alteration type.

A missense single-nucleotide variant (SNV) can lead to an SAV, which is an alteration in the protein sequence. Missense variants that encode a single change in the amino acid sequence of an affected protein represent around 45% of the known disease variants associated with cancer [31–33]. SNVs can be synonymous, non-synonymous or stop gain change. Each type alters the function of the protein differently. Indels and structural variations are also variants that result in altered protein function. Another division of the variants type is based on the type of cells, which can be either germline or somatic [34]. The types of variants are summarized in Figure 1.

**Figure 2:**
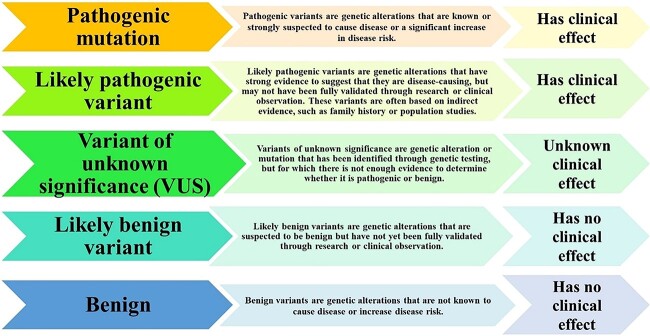
The classification of variants, their definition and their clinical effect.

The distinction of a pathogenic SAV from a benign SAV is critical for improving knowledge of the relationship between genes and diseases in the post-genomic age and facilitating the identification of innovative treatment methods for complex disorders. The accurate classification of a genetic variant effect on diseases is challenging to attain regardless of the abundance of the accumulated genetic variants data over the past few decades. Most existing functional impact prediction software for amino acid changes considers that protein sequences have survived natural selection among recognized living species. As a result, evolutionarily conserved amino acid locations across various species are considered functionally significant, while those found at conservation sites are considered to be harmful [35]. As per the ACMG guidelines, variants are classified into five categories based on their clinical effects. The classification of variants, their definition and their clinical effects are shown in Figure 2.

## BREAST CANCER

One of the most common tumors in the world and accounting for around 11% of all cancer mortality cases in women between 20 and 50 years is BC [5]. For women worldwide, it is responsible for more disability-adjusted life expectancy years than any other cancer. In any country, it can occur in women of any age group from puberty onward, and the risk increases with age. Therefore, there is an urgent need for a reliable and accurate system to aid in the early detection and diagnosis of BC.

### BC-related genes and variants

With the advances in technologies, specifically in the genomics area, many BC-associated genes have been identified in oncogenes and anti-oncogenes. Variants and aberrant amplifications are crucial to the development and growth of tumors. Family history and inherited genetic variants are one of the most critical risk factors associated with BC. Some variants in BC-related genes are known to greatly affect the development of BC. On the other hand, many other genetic variants might affect BC but these are not yet clearly understood. Some gene variants are known to highly predispose women to the development of BC with some having penetrance reaching up to 80%. Most available pathogenicity prediction tools developed for BC focus mainly on well-known genetic variants such as *BRCA1*, *BRCA2, TP53* or *PTEN* variants. Training an ML tool on genes associated with BC can improve its prediction accuracy for BC cases. However, this needs to involve all known genes associated with BC development. Table 1 below summarizes currently well-known genes associated with BC that can be used as training data when developing a BC-specific pathogenicity prediction tool.

**Table 1:** Examples of genes associated with BC and their functions.

Gene	Full name and Location		Classification	Function	Abnormality after mutation	Reference
*BRCA1*	Breast Cancer 1 17q21		Tumor suppressor gene	Plays a role in DNA repair and maintenance of genomic stability.	Impairs the DNA repair function.	[36, 37]
*BRCA2*	Breast Cancer 2 13q12		Tumor suppressor gene	Plays a role in DNA repair, particularly in the repair of double-strand breaks.	Impairs the DNA repair function.	[38, 39]
*HER2*	Human Epidermal Growth Factor Receptor 2 17q12		Oncogene	Encodes a protein involved in cell growth and division.	Leads to uncontrolled cell growth and division.	[40]
*EGFR*	Epidermal Growth Factor Receptor 7p12		Oncogene	It is involved in cell growth and division.	Mutations cause constitutive activation of the EGFR receptor, leading to uncontrolled cell growth, division and progression of BC.	[41, 42]
*c-Myc*	Myc proto-oncogene protein8q24		Oncogene	Plays a critical role in the regulation of cell growth, differentiation and apoptosis.	Leads to dysregulated cell growth, impaired differentiation and decreased apoptosis, contributing to the development and progression of BC.	[43, 44]
*Ras*	Rat Sarcoma viral oncogene homolog- (Harvey) H-Ras - 11p15 (Kristen) K-Ras - 12p12 (Neuroblastoma) N-Ras - 1p22		Oncogene	Encode GTPases that play important roles in normal cell growth, differentiation and survival.	Leads to constitutive activation of the Ras protein, resulting in uncontrolled cell growth, impaired differentiation and resistance to apoptosis.	[45]
*TP53*	Tumor Protein 53 17p13.1		Tumor suppressor gene	Plays a critical role in maintaining genomic stability and preventing the development of cancer by promoting cell cycle arrest, DNA repair and apoptosis.	Leads to the accumulation of genetic damage and promoting the development and progression of BC.	[46, 47]
*NME1*	NME/NM23 nucleoside diphosphate kinase 1 17q21.3		Tumor suppressor gene	Plays a critical role in inhibiting tumor invasion and metastasis through its involvement in nucleotide metabolism, cell migration and signaling pathways.	Leads to the development and progression of BC by promoting tumor invasion and metastasis and is associated with a poorer prognosis.	[48, 49]
*RB1*	Retinoblastoma 1 13.2		Tumor suppressor gene	Regulates cell cycle progression, differentiation and apoptosis by controlling the activity of E2F transcription factors and other downstream targets.	Leads to uncontrolled cell proliferation, impaired differentiation and resistance to apoptosis.	[50, 51]
*PTEN*	Phosphatase and Tensin Homolog 10q23.3		Tumor suppressor gene	Regulates cell growth, proliferation and survival by negatively regulating the PI3K/Akt signaling pathway and promoting apoptosis and cell cycle arrest.	Leads to constitutive activation of the PI3K/Akt pathway, promoting uncontrolled cell growth, proliferation and survival.	[52, 53]
*ATM*	Ataxia Telangiectasia Mutated 11q22-q23		Tumor suppressor gene	Plays a critical role in detecting and repairing DNA damage, promoting cell cycle arrest and inducing apoptosis in response to genotoxic stress.	Impair the ability of cells to respond to DNA damage, leading to genomic instability and an increased risk of developing BC.	[54]
*CDH1*	Cadherin 1 16q22.1		Tumor suppressor gene	Encodes the E-cadherin protein, which plays a critical role in maintaining cell–cell adhesion, polarity and tissue architecture and regulating cell proliferation and differentiation.	Leads to reduced cell adhesion, impaired tissue integrity and enhanced cell motility and invasion.	[55]
*FHIT*	Fragile Histidine Triad 3p14.2		Tumor suppressor gene	Plays a critical role in regulating cell proliferation, DNA damage response and apoptosis, by promoting the cleavage of diadenosine triphosphate (Ap3A) and inhibiting signaling through the Wnt/β-catenin pathway.	Impair the ability of cells to respond to DNA damage and undergo apoptosis, promoting uncontrolled cell growth.	[56]
*Maspin*	Serpin Family B Member 5 18q21.33		Tumor suppressor gene	Regulates multiple cellular processes, including cell adhesion, migration, invasion, angiogenesis and apoptosis, by modulating signaling pathways involving integrins, growth factors and transcription factors.	Promote tumor growth, invasion and metastasis and is associated with a poorer prognosis in BC.	[57, 58]
*PIK3CA*	Phosphatidylinositol-4,5-Bisphosphate 3-Kinase Catalytic Subunit Alpha 3q26.3		Oncogene	Encodes the p110α subunit of phosphatidylinositol 3-kinase (PI3K), a critical signaling molecule that regulates cell growth, survival and metabolism, by activating the AKT/mTOR pathway and other downstream effectors.	Activate the PI3K pathway, leading to uncontrolled cell proliferation, survival and invasion.	[50]
*CCND1*	Cyclin D1 11q13		Oncogene	Encodes cyclin D1, a protein that promotes cell cycle progression by activating cyclin-dependent kinases and facilitating the transition from G1 to S phase and also has non-cycling functions in transcriptional regulation, cell migration and apoptosis.	Overexpression or amplification of CCND1 can drive excessive cell proliferation, survival and invasion.	[59]

### Relevant genetic and genomic variants databases

Recent advances in the field of molecular biology, coupled with the increased affordability of its associated techniques, have paved the way for the study of biological parameters, novel organisms and pathogens, as well as genetic diseases through the sequencing of genetic material. The vast amounts of data generated by these methods necessitate a high degree of expertise and computational power to process, identify and classify genetic variants that may provide scientifically valuable insights. Genomic studies have allowed us to uncover critical information and gain a better understanding of the molecular mechanisms underlying both our biology and various genetic diseases. By starting with the sequencing of small segments of genetic material and moving on to disease-specific gene panels and, more recently, whole exome and genome sequencing, we can, in some cases, trace the origins of a disease, enabling targeted therapy and significantly impacting the clinical decisions made for affected patients or their families [60, 61].

These studies allowed the creation of several databases and beyond, like The Cancer Genome Atlas (TCGA) [62], ClinVar [20] and The Catalogue of Somatic Mutations in Cancer (COSMIC) [19] and others, which provide us the curated data of the molecular alterations related to diseases and serve as a deposit for new studies. All these databases are major contributors to past and new studies and support variant classification [30, 63]. In 2015, several parameters were proposed by the ACMG [30] to be used to evaluate the pathogenicity of germline variants and one of the most widely applicable parameters is *in silico* analysis. This same analysis is also included in the guidelines for somatic variants as recommended in 2017 by the Association for Molecular Pathology, the American Society of Clinical Oncology and the College of American Pathologists [63] and more recently, in 2022, by the Clinical Genome Resource (ClinGen), Cancer Genomics Consortium (CGC) and Variant Interpretation for Cancer Consortium (VICC) [64].

**Figure 3:**
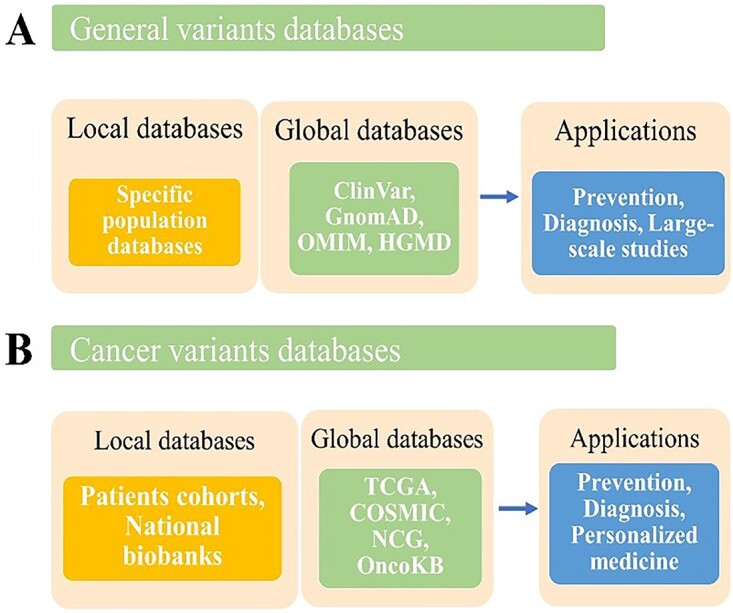
Examples on cancer and general variants databases and applications.

Human variant databases usually have a specific scope and associated content. They can be used for predicting diseases [16] through supporting personalized medicine [17]. These databases have various limitations, including data structure compatibility and the variety of the data they hold in general. As a result, acquiring detailed information on a variation of interest is difficult [22]. Although use of several resources to analyze variant data has been explored [23], the data integration itself is largely for targeted tools and pipelines. Training and testing data are the most crucial elements for the success of any ML tool. The better the data used, the better the outcome. Different variant databases have different structures and datasets within them. Depending on the aim, the most appropriate database must be chosen. Figure 3 shows some examples of variants databases and their applications. The databases that are commonly used for the BC variant pathogenicity prediction tools are discussed below.

#### Human Gene Mutation Database (HGMD)

The Human Gene Mutation Database (HGMD) was initiated in 1996. It aimed to support the clinical study of variations in human genes underlying genetic diseases [65, 66]. The HGMD aims to compile all known genetic variations that cause inherited disorders that have been reported in peer-reviewed journals including clinical genetic laboratories research. Over the last two decades, it has steadily gained a far more significant value as the principal unified repository for disease-related genetic germline variants. It has, for example, been used to enhance cancer prediction in high-risk hereditary BC families [67]. The HGMD provides a comprehensive set of published germline variants in genes that are thought to underlie or are closely associated with human-inherited disease. At the time of writing (December 2022), the HGMD comprised 234 987 publicly identified variants, with 117 744 privately identified variants from the *HGMD Professional 2021.4*. During the CAGI5 ENIGMA challenge, Color Genomics submitted four prediction sets with Learning from Evidence to Assess Pathogenicity (LEAP) [68, 69], an ML tool that predicts variant pathogenicity according to features including datasets from the HGMD and GnomAD databases. The overall performance accuracy achieved by LEAP was 83% [69].

#### ClinVar

ClinVar [70] is a free public human genetic variant collection comprising interpretations of their significance to diseases. It was released in 2013. The National Centre for Biotechnology Information (NCBI) maintains ClinVar within the National Library of Medicine (NLM) at the National Institutes of Health (NIH). Clinical testing laboratories, research laboratories, locus-specific databases, expert panels and other groups submit clinical significance information of variants or sets of variants to ClinVar [70]. ClinVar data were applied to a study by Metin and Pemra [71] to assess the performance metrics of *in silico* pathogenicity methods on functional relevance of cancer variants obtained from ClinVar. They examined the pathogenicity predictions of cancer-related variant datasets of eight cancer types including BC retrieved from ClinVar using 13 different *in silico* tools. A combination of statistical performance metric analysis, prediction distribution frequency data and ROC curve analysis results have suggested that among all *in silico* prediction tools, the top three tools with the highest discriminatory power were found to be MutPred (AUC = 0.677), MetaSVM (AUC = 0.645) and Revel (AUC = 0.637). ClinVar data were applied also in Lin *et al*. [72], where they identified *BRCA1* VUSs from clinical sequencing data and wanted to interpret the clinical significance of such data. Several ML methods have been created to estimate the pathogenic hazards of variations of unknown significance. An optimized random forest algorithm outperformed the performance after benchmarking, and it was selected to predict *BRCA1* VUSs from both the generated sequencing data and ClinVar data. A predicted pathogenicity of 6322 VUSs was obtained, of which 1593 variants were predicted to be pathogenic and 4729 were predicted to be benign [72].

#### Catalogue Of Somatic Mutations In Cancer (COSMIC)

The Catalogue Of Somatic Mutations In Cancer (COSMIC) [19], launched in 2004, offers a collection of somatic variant data from various public sources through one standardized repository that makes it easy to be explored in various ways. COSMIC includes all forms of human cancers, from the most frequent to the extremely rare cancers, observed by clinicians possibly once or twice in a career. Data within COSMIC are collected from scientific publications of clinical, genetic and cancer-related research. COSMIC has developed into a large genome-wide system to investigate patterns of somatic variants in all cancer types. Moreover, recent studies have characterized specific variants in the evolution of genetic resistance to clinical therapeutics. The implementation of FATHMM-MKL (designed based on the characteristics of germline non-cancer variants) for predicting the pathogenic status of cancer somatic variants in the COSMIC dataset has shown good pathogenicity prediction results for BC [73, 74].

#### The Cancer Genome Atlas (TCGA)

The Cancer Genome Atlas (TCGA) [62] and the International Cancer Genome Consortium (ICGC) were launched as the two major projects in 2005 and 2008, respectively. They were developed to use innovative genomic technologies including single-cell sequencing, whole genome and whole exome sequencing to improve the understanding of cancer genetics and create new methods of cancer treatment, diagnosis and prevention strategies. The National Institutes of Health initiated the TCGA Pilot Project to compile a comprehensive atlas of cancer genomic profiles. The TCGA is a public effort that intends to catalog and detect significant cancer-causing genomic changes in large cohorts of over 30 human malignancies utilizing modern genome sequencing techniques and integrated multi-dimensional analysis. These publicly available cancer genetic databases enable the advancement of diagnostic technologies, treatment guidelines and support [62, 75]. In a recent study, a total of 80 227 somatic SNVs from 976 patients were analyzed and the genomic features for 8647 somatic SNVs from 142 young patients (<45 years old at diagnosis) were identified. The data collected from the TCGA database included 6910 somatic SNVs from coding regions and 1737 somatic SNVs from non-coding regions of the genome [76].

### The Genome Aggregation Database (GnomAD)

The Genome Aggregation Database (gnomAD) [77] is one of the leading and most widely used collections of variants from synchronized sequencing data. To support quick and automatic variant analysis, the data are accessible through the online gnomAD browser. The Exome Aggregation Consortium (ExAC) dataset, the first significant compilation of existing sequence data from 60 000 individuals, was published in 2014 [78]. Mainly, gnomAD is generated using whole genome and whole exome sequencing data in addition to single-cell sequencing technologies. ExAC was renamed gnomAD after genome data were added, and it now contains variant data from more than 195 000 people. With more than 150 000 weekly page views, it is currently the most used reference population dataset. Using a non-Finnish non-cancer European population dataset as their control dataset, Rofes *et al*. [79] downloaded and filtered variants to identify predicted loss-of-function variants in *BRCA1*-*associated ring domain 1* (*BARD1*). Copy number variants screening was performed on the gnomAD SVs v2.1 dataset. This study showed results that support the role of *BARD1* as a moderate-penetrance BC-predisposing gene and highlighted a strong association with triple-negative tumors [79].

### Network of Cancer Genes (NCG)

The Network of Cancer Genes (NCG) [80] is a comprehensive database released in 2010 that gathers a collection of curated cancer genes from cancer transcriptomic sequencing screens including next-generation sequencing, single-cell sequencing, whole exome and whole genome sequencing. The NCG is a freely available, manually curated repository of 2372 genes whose somatic modifications have known or predicted cancer driver roles. In 2018, the project reached its 6th release. The NCG genes were collected from 275 articles; 2 included known cancer genes and 273 included cancer sequencing screens from 34 905 cancer donors and various primary locations, covering more than 100 cancer types. In comparison to the previous version, this represents a content increase of more than 1.5-fold. Additionally, NCG annotates characteristics of cancer genes like duplicability, evolutionary origin, RNA and protein expression, interactions between miRNA and proteins and protein function and essentiality. The data from this database were not found in any pathogenicity prediction research, so it represents an exciting opportunity for the future [81].

### Online Mendelian Inheritance in Man (OMIM)

Online Mendelian Inheritance in Man (OMIM) [82] is a comprehensive and authoritative knowledge base of human genes and genetic disorders compiled to support human genetics research and education and support the practice of clinical genetics. It includes data from genome-wide association studies, next-generation sequencing, Sanger sequencing and others. OMIM is now distributed electronically by the NCBI. The Entrez suite of databases is combined with OMIM. Written and edited at Johns Hopkins University with input from scientists and doctors worldwide, OMIM is derived from biomedical literature. Each OMIM entry includes a full-text summary of a genetically determined phenotype and/or gene, as well as numerous links to other genetic databases, such as those for DNA and protein sequence, PubMed citations, general and locus-specific variant databases, HUGO nomenclature, MapViewer, GeneTests, patient support groups and a lot more. OMIM provides a gateway to the rapidly expanding body of knowledge in human genetics. OMIM also has datasets on most cancer types, including BC that has not yet been used in any pathogenicity prediction tool training or testing.

### IntOGen-mutations

IntOGen-mutations [83] provides a resource for locating cancer drivers among various tumors that were identified using functional genomic analysis, whole exome and whole genome sequencing and so on. It can display the findings of the most recent large tumor somatic variant data sets that have undergone systematic analysis. It focuses on copy-number gains and losses and transcriptomic changes in tumors. The outcomes of tumor genome analyses conducted using various variant-calling workflows are integrated into the IntOGen-mutations database. To thousands of tumor genomes, it is scalable. Without the need to estimate the background variant rate, it offers a tool that identifies genes predisposed to accumulating variants with high functional effects. It also provides a tool that detects genes whose variants are highly functionally significant. Both tools look for signs of positive selection seen in genes whose variants are potential drivers of tumor formation. IntOGen-mutation data have not yet been used in research related to predicting the pathogenicity of BC-causing variants.

### cBio Cancer Genomics Portal (cBioPortal)

The open-source cBio Cancer Genomics Portal (cBioPortal) is a tool for viewing multi-dimensional cancer genomics data sets interactively [84]. It includes single-cell sequencing, whole exome and whole genome sequencing and other functional genomic assays data. Although open-source, germline datasets are not publicly accessible [85]. The cBioPortal has access to data from over 5000 tumor samples from 20 cancer studies [84]. The cBio Cancer Genomics Portal removes considerable barriers between complex genomic data and cancer researchers that want rapid and easy access to molecular profiles and clinical features from large-scale cancer genome studies. It helps researchers to get biological insights and clinical information by utilizing these large data sets. There are 15 initial TCGA data sets and 5 published data sets accessible on the cBioPortal. Based on the most recent TCGA production runs, provisional TCGA data sets are updated weekly, and the site is continuously updated when additional TCGA cancer types are introduced. Variant information is present in published data sets but not in tentative data sets. Variant data are made public and uploaded to the site once each cancer type within TCGA is completed and somatic variants are validated. The site also provides information on copy number changes, mRNA expression changes based on microarray and RNA sequencing, DNA methylation values, protein and phosphoprotein levels and variant data.

### DriverDBv2

DriverDBv2 [86] is an updated version of DriverDB. This is a database that includes over 6000 cases of whole exome and whole genome sequencing data, functional genomic assays and published bioinformatics techniques and annotation databases for driver gene/variant identification. The database provides two points of view, ‘Cancer’ and ‘Gene’, to help researchers visualize the connection between cancers and driver genes/variants. In the DriverDBv2 database, over 9500 cancer-related RNA-sequencing datasets and over 7000 exome-sequencing datasets were integrated from TCGA, ICGC and numerous published papers. Seven additional computational algorithms have been developed for driver gene identification and incorporated into the analysis pipeline. Gu *et al.* applied FI-net and 22 other state-of-the-art tools to 31 datasets, including DriverDBv2 [87]. According to their comprehensive evaluation, FI-net outperformed other tools with results illustrating that FI-net could identify known and potential novel driver genes [87].

### OncoKB

OncoKB is an inclusive precision oncology knowledge database released in 2017 [88]. It provides comprehensive, evidence-based oncological somatic variants and structural changes knowledge found in patient tumors to support their therapy choices [88, 89]. It includes data generated through whole exome and whole genome sequencing, proteomics, immunohistochemistry and other functional genetic assays. OncoKB data are managed by a dedicated panel of clinicians and cancer biologists who evaluate and manage biomarker-associated investigational therapeutic strategies. OncoKB connects data on (Food and Drug Administration) FDA-approved treatments and investigational drugs undergoing clinical trial evaluation for biomarker-guided use. Additionally, it emphasizes unfavorable clinical findings to discourage the off-label use of costly targeted therapies that have been demonstrated to be ineffective in particular variational contexts. An interactive website and the cBioPortal for Cancer Genomics both offer access to OncoKB. By assisting doctors in finding potentially actionable variants to ensure that patients receive the proper remedies or are directed to the most pertinent clinical trials, a curated database like OncoKB can play a crucial role in helping to realize the promise of precision medicine [88].

### Functional Annotation of Somatic Mutations in Cancer (FASMIC)

Functional Annotation of Somatic Mutations in Cancer (FASMIC) [90] is a user-friendly, interactive and open-access web platform for comprehensive visualization and exploration of variant-associated data [90] collected from different genomic functional assays including next-generation sequencing, whole exome and whole genome sequencing. It includes modules such as brief description, 3D structures, literature, variant frequency, functional prediction and protein expression. To find a variant, users can first query its gene symbol and select the matched genes to show all related variants. All variations investigated are displayed in a tabular style, together with critical information for each variant, such as gene name, chromosomal location, amino acid change and functional annotation. A Function Prediction module gives function predictions generated by well-known computational techniques. Furthermore, a Protein Expression module provides extensive protein expression data of cell lines affected by variations compared with wild-type genes. This aids in understanding the unique functional effects of variations.

### Cancer Cell Line Encyclopedia (CCLE)

The Cancer Cell Line Encyclopedia (CCLE) [91] is a collection of 947 human cancer cell lines’ genomic functional assays including whole exome and genome sequencing, gene expression, genomic copy number and massively parallel sequencing big-scale genomic datasets, as well as pharmacologic assays of 24 drugs across over 500 of these lines [91]. The CCLE encompasses 36 tumor types with several genomic technology platforms used for characterizing cell lines. The variational status of over 1600 genes was determined by targeted massively parallel sequencing, followed by removal of variants likely to be germline events. 392 recurrent variants affecting 33 known cancer genes were assessed by mass spectrometric genotyping. DNA copy number was measured using a high-density single-nucleotide polymorphism array. Eventually, mRNA expression levels for each of the lines were determined. These results were also utilized to validate cell lines. In a drug response prediction study and through leave-one-out cross-validation and cross-classification on independent datasets, it was shown that using this dataset for prediction leads to an accurate and reproducible classification of sensitive and resistant cell line–drug pairs with a high degree of accuracy [92].

### Comparison

Several available databases have been developed for cancer-causing gene identification. The differences in the data structures and nature of the data types in each database along with diversity of curation information give different results when comparing these resources using ML tools. Moreover, some databases like the CCLE, COSMIC and others demonstrate functional information regarding the variant and its effect on the interaction of the drug with its ligand; based on that, personalized treatment for each patient’s variant can be established. The personalized treatment can be either a new drug or natural product that is found to bind perfectly to the mutated ligand or repurposed drug, which is any FDA-approved drug that was not initially indicated to treat the disease but is found to be perfectly act on the mutated ligand. Our goal is to choose the most suitable data sources for a given tool to predict the pathogenicity of variants. Some databases were not previously used to train or test any BC pathogenicity prediction tools; however, they are good candidates for future BC-specific tools training and testing. Table 2 summarizes the databases of variants, the variant type and the accessibility and the location (website) of each database. Additionally, an application of different cancer-related databases in BC pathogenicity prediction is provided in Table 3 with the advantages and disadvantages of each database summarized in Table 4.

**Table 2:** The summary of the databases of cancer-related variants, the variant type, the accessibility and the location of each database.

Database	Full form name	General description	Targeted disease	Variants type	Website
HGMD	Human Gene Mutation Database	Is a repository of inherited variant data useful for medical research, genetic diagnosis and next-generation sequencing studies.	General	Somatic and germline	https://www.hgmd.cf.ac.uk/ac/index.php
ClinVar	Clinical significance variants	Is a freely available archive for interpretation of the clinical significance of variants for reported conditions.	General	Somatic and germline	https://www.ncbi.nlm.nih.gov/clinvar/
COSMIC	Catalogue of Somatic Mutations in Cancer	Is a database of information about somatic variants in cancer obtained from curating relevant literature and high-throughput sequencing data generated by the Cancer Genome Project and others.	Cancers	Somatic	https://cancer.sanger.ac.uk/cosmic
TCGA	The Cancer Genome Atlas	Is a project to identify the complete set of DNA changes in many different types of cancer.	Cancers	Somatic and germline	https://www.cancer.gov/about-nci/organization/ccg/research/structural-genomics/tcga
GnomAD	The Genome Aggregation Database	Is a resource developed by an international coalition of investigators to aggregate and harmonize exome and genome sequencing data from a wide variety of sequencing projects with summary data for the broader scientific community.	General	Germline	https://gnomad.broadinstitute.org/
NCG	Network of Cancer Genes	Is a manually curated repository of genes whose somatic modifications have known or predicted cancer driver roles.	Cancers	Somatic	http://ncg.kcl.ac.uk/
OMIM	Online Mendelian Inheritance in Man	Is a continuously updated catalogue of human genes and genetic disorders and traits, with particular focus on the gene–phenotype relationship.	General	Allelic	https://www.omim.org/
IntOGen	Integrative Onco Genomics	Is a framework for automatic and comprehensive knowledge extraction based on variant data from sequenced tumor samples. The framework identifies cancer genes and pinpoints their putative mechanism of action across tumor types.	Cancers	Somatic	https://www.intogen.org/about
CBioPortal	The cBio Cancer Genomics Portal	Is an exploratory analysis tool for exploring large-scale cancer genomic data sets from large consortium efforts, like TCGA, as well as publications from individual labs.	Cancers	Somatic and germline	https://www.cbioportal.org/
DriverDB	Driver Database	Is a cancer omics database that integrates somatic variants, RNA expression, miRNA expression, methylation, copy number variation and clinical data with annotation and published bioinformatics algorithms.	Cancers	Somatic	http://driverdb.tms.cmu.edu.tw/
OncoKB	Oncology Knowledge Base	Is a comprehensive and curated precision oncology knowledge base that offers oncologists detailed, evidence-based information about individual somatic variants and structural alterations in patient tumors with the goal of supporting optimal treatment decisions.	Cancers	Somatic	http://oncokb.org
FASMIC	Functional Annotation of Somatic Mutations in Cancer	Is a comprehensive database for understanding the functional impact of somatic variants in cancer. It provides functional annotations with protein expression, variant frequency, 3D structures, function prediction and literature to help researchers explore variant details.	Cancers	Somatic	https://bioinformatics.mdanderson.org/public-software/fasmic/
CCLE	Cancer Cell Line Encyclopedia	Is a database of gene expression, genotype and drug sensitivity data for human cancer cell lines.	Cancers	Somatic and germline	https://sites.broadinstitute.org/ccle/datasets

**Table 3:** The application of different cancer-related databases in BC pathogenicity prediction.

Database	Application example	Reference
HGMD	Color Genomics by Lai *et al.* submitted four sets of predictions using LEAP, a machine learning framework that predicts variant pathogenicity according to features based on training datasets from the HGMD.	[68]
ClinVar	Lin *et al.* identified *BRCA1* VUSs from clinical sequencing data. 1593 VUSs were predicted to be pathogenic, and 4729 VUSs were predicted to be benign. Yazar *et al.* used a combination of statistical performance metric analysis, prediction distribution frequency data and ROC curve analysis results have suggested that among all *in silico* prediction tools, the top three tools with the highest discriminatory power were found to be MutPred (AUC = 0.677), MetaSVM (AUC = 0.645) and Revel (AUC = 0.637).	[71, 72]
COSMIC	FATHMM-MKL was used for predicting the pathogenic status of cancer somatic variants in the COSMIC dataset. It was shown to have good prediction results for BC pathogenicity.	[73]
TCGA	Feizi *et al.* applied various models to predict the pathogenic status of somatic variants identified in young BC patients from TCGA-*BRCA* studies. The results indicated that using their model predicted 1853 positive SNVs (out of 6910) from the TCGA-*BRCA* dataset.	[76]
GnomAD	Rofes *et al.* used the gnomAD non-Finnish European population, non-cancer dataset as a control population for their study. This study showed results that support the role of *BARD1* as a moderate-penetrance BC-predisposing gene and highlight a strong association with triple-negative tumors.	[79]
DriverDB	Gu *et al.* applied FI-net and other 22 state-of-the-art tools to 31 datasets including DriverDBv2. According to the comprehensive evaluation, FI-net outperformed the other tools. Furthermore, the results illustrated that FI-net could identify known and potential novel driver genes.	[87]
CCLE	In a drug-response prediction study and through leave-one-out cross-validation and cross-classification on independent datasets, it was shown that using this dataset in the prediction leads to accurate and reproducible classification of sensitive and resistant cell line–drug pairs with a high degree of accuracy.	[92]

**Table 4:** The advantages and disadvantages of the presented databases.

Database	Advantages	Disadvantages
HGMD	Comprehensive for all disease-causing variants. Provides variant-specific links to several other databases.	Includes only a single reference for each variant. Includes only disease-causing variants for general diseases.
ClinVar	Comprehensive for all known disease-causing and non-disease-causing variants.	Includes variants regardless of association with disease. Provide access to all observed variants but may not be supported by peer-reviewed literature.
COSMIC	Accurate and consistent data. Actionability functionality allows users to search drugs that target somatic variants at all stages of drug development, including those still in development, in clinical trials or that have been repurposed.	Manually curated, which is time-consuming and not rapidly modified.
TCGA	Provides a large number of cancer-specific samples. Offers multiple data platforms for the same sample. Offers unified data generation and low-level analysis.	The clinical data are spotty as almost all the samples are primarily untreated, without any response data and short follow-up. There are no immune-oncology data. Samples in the TCGA project are all fresh frozen samples, which are not commonly used in clinical settings.
GnomAD	GnomAD’s predecessor, the Exome Aggregation Consortium (ExAC) database, lies in capturing sequencing data representing diverse European and non-European ancestries at a larger scale compared with previous sequencing studies.	Many populations are underrepresented. Some variants are somatic clonal variants. Not everyone in gnomAD is healthy and young.
NCG	It has cancer-specific variants. It incorporates information about genes with a known or anticipated significance as cancer drivers (predisposition).	It requires the use of *ad hoc* tools for data organizing and mining.
OMIM	Continuously updated. It unravels the complex relationships between genes and disease.	Only few non-protein-coding genes variants are included.
IntOGen	It has cancer-specific variants.	Only contain somatic variants.
CBioPortal	It integrates multiple cancer genomics projects. It enables the users to analyze complex data sets and translate into biologic insights and immediate clinical applications.	It has potential bias to estimate the relative proportion of germline variants, *de novo* variants and rare mutated alleles in a sample.
DriverDB	It incorporates large-scale data mining using many algorithms and then presents summarized driver genes with different kinds of aspects for variant visualization.	It uses tools like SIFT and PolyPhen to calculate scores, although they are not cancer-specific tools, so the results might not be reliable.
OncoKB	It is oncologist-oriented with evidence-based information about individual somatic variants and structural alterations present in patient tumors to support optimal treatment decisions.	As it oncologist specialized, other users might not understand the data.
FASMIC	It provides a comprehensive database for functional impact of somatic variants in cancer.	It does not cover germline variants.
CCLE	It includes data on gene variants, RNA splicing, DNA methylation, histone H3 modification and microRNA expression.	The effects of variant in the cell line and in humans might be different.

## ML TOOLS FOR PREDICTION OF BC PATHOGENICITY

**Figure 4:**
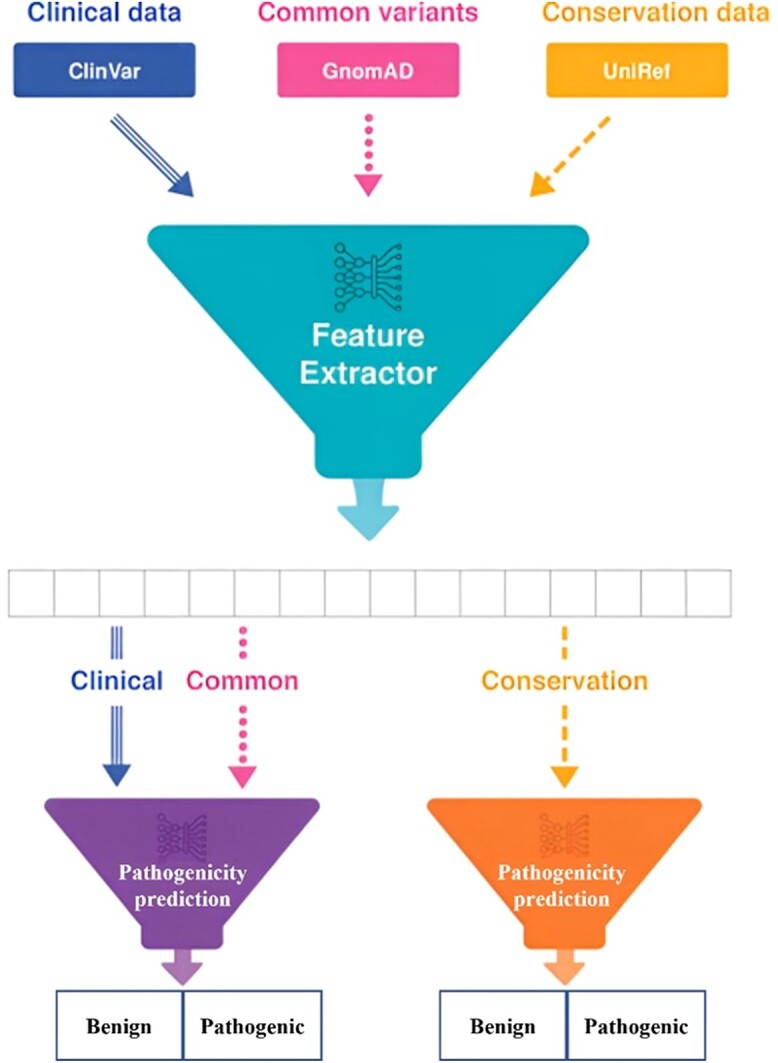
Applying ML in the pathogenicity prediction research. This figure was modified from Won *et al*. [96].

**Figure 5:**
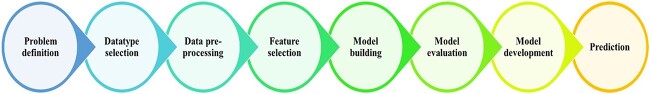
The main workflow of the pathogenicity prediction research using ML.

The ‘gold standard’ prediction of BC pathogenicity as per the ACMG guidelines involves screening procedures consisting of clinical evaluation, radiological imaging and pathological testing [93]. Due to the fact that the traditional gold standard of classification is expensive, human invasive and intensive, some highly accurate prediction tools like SIFT can be used to help in pathogenicity classification. Additionally, new ML tools can be used to serve a similar purpose based on model creation and extensive training and validation. In the training and testing stage, a given ML model makes predictions using input data comprising known/confirmed BC pathogenicity data and benign data [94]. Pre-processing, feature selection and extraction and classification are key elements of ML [95]. The feature extraction part of an ML tool is crucial for cancer diagnosis and prediction. The workflow of the pathogenicity prediction research using ML is shown in Figures 4 and 5.

Many ML tools have been developed and applied to predict the potential pathogenic effect of variants. Some of these tools were developed explicitly for given diseases, while others have been developed to be general purpose. In this work, we consider 14 ML-based and 2 non-ML-based pathogenicity prediction tools, which we discuss below. The non-ML tools were added to be able to compare between the ML- and non-ML-based tools performance. We provide a description of each tool, the type of application, the advantages and disadvantages, the algorithm used in the development of the tool and the reliability and tool links for each of the tools.

### Combined Annotation-Dependent Depletion (CADD)

Combined Annotation-Dependent Depletion (CADD) [97] is a free, commonly used pathogenicity prediction tool that uses a logistic-regression ML model to categorize causal variants in genetic analysis, with a specific focus on highly penetrant contributors to severe Mendelian disorders. It was originally trained on various datasets from different databases including gnomAD, ClinVar and others. It offers an integrative annotation built from more than 60 genomic features and can score SNVs and short insertions and deletions anywhere in the reference assembly. The ML model CADD uses is trained on a binary distinction between simulated *de novo* variants and fixed variants in humans. The utility of the CADD score was recently reported to rank pathogenicity as C-scores ranging from 1 to 99 for deleterious variants. Using C-scores, Nakagomi *et al.* attempted to constitute a classification system for *BReast CAncer genes* (*BRCA*) *1* and *2* variants of uncertain significance. It was found that CADD can classify *BRCA 1* and *2* variants and select patients for further segregation studies [97].

### Polymorphism Phenotyping v2 (PolyPhen-2)

Polymorphism Phenotyping v2 (PolyPhen-2) [98] is an ML tool that is used to predict the possible impact of amino acid substitutions on the structure and function of a human protein. It was trained on a variation of databases including UniProt, NCBI RefSeq, sequence alignment and others. It uses a combination of physical and comparative considerations to make predictions. PolyPhen-2 uses eight sequence-based and three structure-based predictive features to predict the effect of a mutation on protein function. These features are selected automatically by an iterative greedy algorithm, which iteratively selects the features that improve the prediction accuracy the most. The algorithm is designed to consider both the overall accuracy of the predictions and the balance between sensitivity and specificity. The distance between the protein containing the first variation from the human wild-type allele and the human protein and whether the mutant allele originated at a hypermutable site are the characteristics that characterize how well two human alleles fit into the pattern of amino acid replacements within the context of multiple sequence alignment of homologous proteins. Using a clustering algorithm, the alignment pipeline chooses the set of homologous sequences to be examined before building and fine-tuning their alignments [98]. The functional significance of an allele replacement is predicted from its individual features based on a Naïve Bayes classifier. In terms of accuracy, [99, 100] reported the performance of PolyPhen-2 for predicting the functional effects varied across a clinical dataset of *BRCA1* and *BRCA2* missense variants. The absence of consistency in prediction outcomes limit the clinical application in classifying pathogenic VUSs identified through molecular testing of *BRCA1* and *BRCA2* [101].

### Fathmm-MKL

Fathmm-MKL [74] is an ML tool that is used to predict the functional effects of missense variants in a protein by combining sequence conservation within hidden Markov models (HMMs), indicating the alignment of homologous sequences and conserved protein domains. Pathogenicity weights are used for the overall tolerance of the protein to variants. Fathmm-MKL is trained on an integration of databases including functional annotations from ENCODE with nucleotide-based sequence conservation measures when assessing the functional consequences of coding and non-coding variants in addition to others. It was observed that Fathmm-MKL had improved performance when compared with other algorithms like CADD when predicting the functional impact of SNVs [74]. Nono *et al.* have shown that Fathmm-MKL effectively predicted the pathogenicity of BC-causing gene variants with a Pearson’s correlation coefficient of 0.80, outperforming other tools used in that research [73].

### Rare Exome Variant Ensemble Learner (REVEL)

Rare Exome Variant Ensemble Learner (REVEL) is an ML ensemble tool used for predicting the pathogenicity of missense variants based on several other tools including MutPred, FATHMM, VEST, PolyPhen, SIFT, PROVEAN, MutationAssessor, MutationTaster, LRT, GERP, SiPhy, phyloP and phastCons. REVEL was trained with recently discovered pathogenic and rare neutral missense variants and excluded those used previously in training the original (individual) tools; this makes up the huge volume of the data used to train REVEL overall. REVEL performed very well in predicting the pathogenicity of variants compared with individual tools [102]. Although REVEL was not initially developed for predicting BC pathogenic variants, it has shown good performance with an area under the curve (AUC) of 0.79, which is one of the highest accuracy values compared with tools not designed specifically for BC [103].

### CScape

CScape [104] is an ML–based tool for predicting the probability of a variant to drive cancer. It was trained using datasets from COSMIC and 1000 Genomes Project databases. CScape outperforms alternative tools on somatic variants, reaching 91% accuracy in coding regions and 70% in non-coding regions. Using thresholds to separate high-confidence predictions can increase accuracy. A statistical method was used to distinguish the coding from the non-coding regions of the cancer genome, which tends to cluster in genomic regions where optimistic predictions are made to distinguish between recurrent and rare variants in the human cancer genome in advance [104]. CScape-somatic [105] is an integrative classifier tool that is used to predictively discriminate between recurrent and rare variants in the human cancer genome. It was trained on datasets from the COSMIC database and the International Cancer Genome Consortium Data Portal. This tool is designed to work with somatic point variants in both coding and non-coding regions of the genome. It uses only cancer genome data to examine the difference between rarely occurring and frequently occurring somatic single-point variants in the human cancer genome. The authors of this tool have shown that this type of predictive differentiation can offer a fresh perspective and potentially a more precise prediction in both the coding and non-coding regions of the cancer genome. It’s important to note that this tool is focused on somatic mutations, which are mutations that occur in cells that are not germ cells and that are not passed down to the next generation. This is different than germline mutations that are present in every cell of the body and are inherited from a parent. When tested on somatic variants, CScape-somatic outperforms rival tools, achieving balanced accuracy in coding areas of 74% and non-coding regions of 69%. Using thresholds to extract high-confidence predictions can increase accuracy [105].

### DeepDriver

DeepDriver [106] is an ML–based tool based on deep neural networks that performs convolution of variant-based features of genes and their neighbors in similarity networks. It was suggested that similarity networks and attributes that describe the functional impact of variants might be used to determine driver genes. A convolutional neural network trained using a variant-based feature matrix built based on the topological structure of a similarity network specifically predicts putative driver genes. This tool is trained on different datatypes including gene expression data from the NCI Genomic Data Commons and functional annotations from COSMIC. The technique takes advantage of the similarities between gene expression patterns and the functional effects of variants simultaneously. This makes it possible to combine two types of data and increase prediction accuracy. The technology improves the prediction of driver genes by enabling the convolutional neural network to learn information from variant data and similarity networks simultaneously [106].

### DNA-repair Associated Breast Cancer (DrABC)

DNA-repair Associated Breast Cancer (DrABC) [107] is another DL-based tool that enhances the accuracy of identifying germline pathogenic variants (GPVs) carriers in cancer predisposition genes (CPGs). It can locate GPVs and CPGs among BC patient–centered different endophenotypes with GPVs in genes engaged in homologous recombination and other DNA repair pathways. It was trained on a Chinese-specific discovery cohort. Lui *et al.* evaluated a multi-center cohort of 3041 female Chinese BC patients who underwent multi-gene genetic testing. Incorporating the detailed phenotypes of numerous cancer types and their family histories. A phenotype-driven prediction model based on a hierarchical neural network architecture was developed to recognize hereditary BC by considering the distinct endophenotypes linked to various CPGs in BC patients. When used to identify GPV carriers among Chinese BC patients, the model performed better than expected [107]. However, such tools are specific to a single disease instead of dealing with all diseases.

### RENOVO

RENOVO [108] is a computational ML–based tool that uses a random forest algorithm to classify genetic variants as pathogenic or benign based on publicly available information. It is trained on a set of pathogenic and benign variants from the ClinVar database. It has been validated on additional datasets, including unreported variants validated either through expert agreement (ENIGMA) or laboratory-based functional assays of *BRCA1/2*. The tool uses feature classifications based on the same guideline recommendations as other existing tools, but it outperforms these other tools on all datasets. This is important as it provides a validated tool to reduce the fraction of uninterpreted or misinterpreted variants, an unmet need in modern clinical genetics. RENOVO can achieve high performance by using a random forest algorithm. This ML algorithm can learn from large amounts of data and identify complex relationships between input features and output labels. It can help improve the interpretation of genetic variants in the clinical setting, which can help diagnose and manage genetic diseases [108].

### Supervised machine learning framework (SVFX)

Supervised machine learning framework SVFX [109] is an ML–based tool to score the pathogenicity of somatic and germline structural variants (SVs). SVFX was trained on datasets from the Pan-Cancer Analysis of Whole Genomes (PCAWG) Project, Genome Sequencing Program (GSP), ClinVar database, gnomAD and 1000 Genomes Project. SVs play a critical role in many diseases, but limited approaches are available for interpreting and prioritizing these variants [110]. SVs cause more substantial variation in an individual genome at the nucleotide level than other variants. Still, they should be more noticed due to the technical challenges associated with their detection and analysis [110]. To address this challenge, the authors of SVFX developed a new framework that utilizes tissue-specific genomic and epigenomic features to score the pathogenicity of SVs [109]. The framework was trained using SV call sets in diseased and healthy individuals and included genomic, epigenomic and conservation-based features. SVFX was applied to SVs in cancer and other diseases and achieved high accuracy in classifying pathogenic SVs. The predicted pathogenic SVs in cancer cohorts were found to be enriched among known cancer genes and many cancer-related pathways. SVFX is a valuable tool for identifying and interpreting structural variants, which can provide a more comprehensive understanding of the molecular mechanisms of various diseases. It can help identify potential driver mutations in cancer and other diseases, aiding in developing new treatments [109].

### Aljarf *et al*.

Aljarf *et al*. [103] developed an ML–based tool for evaluating the functional impact of single-point missense variants in the *BRCA1* and *BRCA2* genes. The tool uses supervised ML, which is a reliable approach for categorizing missense variants in a gene with given clinical effects. It was trained on evolutionary conservation, missense variant prediction models from dbNSFP, physicochemical properties and changes in post-translational modifications. The tool is designed to be both gene-specific for *BRCA1* and *BRCA2* and also a generic tool for evaluating missense variants in other genes. The authors anticipate that this *in silico* saturation mutagenesis tool will be valid and reliable for detecting variants of uncertain significance (VUS) and providing precise functional estimations for newly discovered variants [103]. Additionally, the enhanced prediction performance of the tool could assist researchers in classifying possible single-nucleotide variants (SNVs) in *BRCA1* and *BRCA2* for further exploration and validation. The tools were validated using 10-fold cross-validation, and the final tool models achieved a Matthew’s Correlation Coefficient of up to 0.98. It is assumed that this predictive tool can be an effective tool for guiding the analysis of newly discovered variants and prioritizing variants for experimental validation. It can provide insights into understanding and interpreting the functional outcomes of missense variants in these genes. This tool can be a valuable resource for researchers and clinicians as it can assist in the identification of potential disease-causing variants in *BRCA1* and *BRCA2* genes, which are associated with an increased risk of breast and ovarian cancer [103].

### MutPred and MutPred2

MutPred is a random forest–based ML tool that depends on sequence, conservation, structural and functional characteristics to predict a variant’s pathogenicity classification [111]. MutPred2 is a neural network ensemble tool with an expanded feature set that has been trained on a much larger and more heterogeneous dataset acquired from HGMD, SwissVar, dbSNP and others [112]. MutPred2 was run based on two approaches: with and without considering gene families in training. These characteristics itemize proteins in the human and mouse genomes at several levels of sequence identity to the protein in which the variant is detected. These features were informally referred to as ‘homolog counts’. The only inputs needed for MutPred and MutPred2 are a protein sequence and an amino acid substitution as input and output scores between zero (benign) and one (pathogenic). Both tools provided accurate predictions for *BRCA1* however MutPred outperformed MutPred2 for *BRCA2*. Both tools performed similarly when the ‘probably benign’ were excluded. This was possibly as a result of selection of the MutPred2 model that included protein-level homolog counts as features [112].

### Learning from Evidence to Assess Pathogenicity (LEAP)

Learning from Evidence to Assess Pathogenicity (LEAP) [68] is an ML-based pathogenicity prediction tool. It was trained on missense variants detected and classified during routine clinical testing at Color Genomics. Manual variant classification uses various underlying data types, such as functional prediction, splice prediction, evolutionary conservation, population frequency, protein domain, co-occurring pathogenic (P/LP) variants and individual and family medical histories. LEAP prioritizes the evidence and weights it according to how it contributes to predictions based on the work of different scientists. LEAP’s prediction performance was assessed with growing evidence based on several model types and evaluations of numerous genes and disease areas. Its value as a tool for clinical interpretation was explored based on the Critical Assessment of Genome Interpretation (CAGI5) ENIGMA Consortium [69] who held a blind prediction challenge. Variations of LEAP placed first, second, third and fourth against competing models that were either published or newly developed. This was the first external validation of LEAP’s performance. Apart from excluding any inputs that are not readily available to the general public, LEAP2 acts as a control and is equal to LEAP1 in terms of pathogenicity estimation including use of data from HGMD. Random forest is used in LEAP3 as opposed to regularized logistic regression. Instead of a two-class model (Benign, Pathogenic), LEAP4 employs a three-class regularized logistic regression model (Benign, VUS, Pathogenic).

### LYRUS

LYRUS [113] is an ML tool based on the XGBoost classifier developed to predict the pathogenicity of SAVs. It was trained based on variants collected from the ClinVar database. Most variants in the human genome come from SAVs. Understanding the genomic architecture of complex diseases can be obtained by identifying pathogenic SAVs. Most methods for predicting the pathogenicity or functional impacts of SAVs rely on either structural or sequencing data. LYRUS combines five sequence-based, six structure-based and four dynamics-based features. Uniquely, LYRUS integrates the variation number, a recently suggested characteristic of sequence co-evolution. The ClinVar database’s dataset of 4363 protein structures corresponding to 22 639 SAVs were used to train LYRUS, and the VariBench testing dataset was used to assess its performance. Performance analysis revealed that LYRUS performed similarly to the most popular variant effect predictors. Six deep mutational scanning datasets for *PTEN* and *TP53* were used to benchmark LYRUS’ performance [113].

### Align Grantham Variation Grantham Deviation (Align-GVGD) 

Align Grantham Variation Grantham Deviation (Align-GVGD) [114] is a freely available, web-based tool that uses the biophysical properties of amino acids and protein multiple sequence alignments to predict where missense variations in important genes will fall on a spectrum from enriched deleterious to enriched neutral. It classifies variants according to the level of cross-species conservation observed for a single missense substitution while considering the biophysical characteristics of the amino acids, and it’s considered a non-ML method [114]. In the study by Tavtigian *et al*., an extension of the Grantham difference (A-GVGD) was used to classify missense variations in the *BRCA1* gene. The method combined two techniques: the co-incidence of unclassified variants with clearly deleterious variants and the use of Grantham differences to analyze most missense variants. The researchers used this approach to distinguish known neutral and deleterious missense variants into distinct sets and classified eight unclassified variants as neutral. This approach can be helpful in determining the functional impact of genetic variations in the *BRCA1* gene, which is associated with an increased risk of BC and ovarian cancer [115].

### Sorting intolerant from tolerant (SIFT)

Sorting intolerant from tolerant (SIFT) [116] is a tool that predicts the deleteriousness of an amino acid substitution to a protein. SIFT was trained originally on lacI, lysosyme and HIV protease amino acid substitutions. It is frequently used to prioritize non-synonymous missense variants. An amino acid change may be tolerated, and the protein still functions normally, but sometimes, the protein might not tolerate a given amino acid change. SIFT categorizes the amino acid change as tolerated or deleterious to the protein’s function. SIFT is categorized under the non-ML tools, as it considers protein conservation with homologous sequences alongside the severity of the amino acid change [116]. In multiple studies, SIFT has been shown to achieve high sensitivity levels in predicting the functional impact of variants in the *BRCA1* and *BRCA2* genes. In Poon [101], it was reported that SIFT achieved 100% sensitivity in predicting *BRCA1* and *BRCA2* variants. Similarly, in Kerr *et al*. [100], it was reported that SIFT had 100% sensitivity in predicting both *BRCA1* and *BRCA2* variants. In Ernst *et al*. [99], it was also confirmed that SIFT had 100% sensitivity in predictions on both *BRCA1* and *BRCA2* variants. It’s worth noting that high sensitivity does not imply high specificity, and it may have a high rate of false positives [99].

### Comparison

Many tools have been developed for pathogenicity prediction based on different algorithms and utilizing different training datasets. These differences give rise to slightly different results when comparing them with the same input dataset. The training datatypes and algorithms used to develop a given tool should be considered when selecting the most suitable tool for a given dataset. Table 5 summarizes the application types, the programming language and the algorithm used to develop each of the aforementioned tools along with the reference for each tool that can be referred to for any additional information needed. Table 6 shows the reference for the reliability and significance of each tool, along with the number of citations from Google Scholar. Table 7 shows the advantages and disadvantages of each tool. Moreover, Table 8 shows the functionality of each tool. Finally, Table 9 shows a comparison between the performance of ML-based tools and non-ML-based tools using the AUC values. The gene-specific model tool that is specialized for BC has shown higher AUC compared with other ML-based tools like polyphen-2 and CADD, which, in turn, have shown higher AUC compared with the non-ML based tool SIFT [117]. Similarly, Lyrus, which is another cancer-specific tool, has shown higher performance in terms of AUC compared with the other ML-based tool Polyphen and the non-ML-based tool SIFT [113]. Additionally, the ML-based tools Revel, CADD and Polyphen have shown higher AUC compared with SIFT when tested on the same dataset of BC variants [71]. The tools Polyphen, Revel and SIFT were also used in another study to assess their performance in predicting BC variants, and the ML-based tools have shown higher AUC compared with SIFT [101, 118].

**Table 5:** The summary of the application type, the programming language and the core algorithm realized by the tool.

Tool	Application type	Programming language	Algorithm	Reference
CADD	Web-based	Python	Logistic regression	[97]
PolyPhen-2	Web-based	Python	Naïve Bayes classifier	[98]
Fathmm-MKL	Web-based	Python	Multiple kernel learning	[74]
REVEL	Web-based	–	Ensemble method	[102]
CScape	Web-based	–	Integrative classifier	[104, 105]
DeepDriver	Downloadable code	Python	Convolutional neural network	[106]
DrABC	Web-based	Python	Deep learning	[107]
RENOVO	Web-based	Python + R	Random forest	[108]
SVFX	Downloadable code	Python	Supervised ML	[109]
Aljarf *et al.*	Private	–	Supervised ML	[103]
MutPred and MutPred2	Web-based	MATLAB	Random forest and neural networks	[112]
LEAP	Private	Python	Logistic regression and random forest	[68]
Lyrus	Downloadable code	Python	XGBoost	[113]

**Table 6:** Significance of each tool.

Tool	Number of citations (from Google scholar as of 5 September 2023)	Link of tool/source code	Reference
CADD	17	http://cadd.gs.washington.edu/. https://github.com/kircherlab/CADD-scripts	[97]
PolyPhen-2	3297	http://genetics.bwh.harvard.edu/pph2/	[98]
Fathmm-MKL	602	http://fathmm.biocompute.org.uk/ https://github.com/HAShihab/fathmm-MKL	[74]
REVEL	1429	https://sites.google.com/site/revelgenomics/about	[102]
CScape	48 16	http://cscape.biocompute.org.uk/ http://cscape-somatic.biocompute.org.uk/	[104, 105]
DeepDriver	67	https://github.com/luoping1004/deepDriver	[106]
DrABC	2	http://gifts.bio-data.cn/#/ https://github.com/zhq921/DrABC	[107]
RENOVO	13	https://bioserver.ieo.it/shiny/app/renovo https://github.com/mazzalab-ieo/renovo	[108]
SVFX	22	https://github.com/gersteinlab/SVFX	[109]
Aljarf *et al.*	3	Private	[103]
MutPred and MutPred2	45	http://mutpred.mutdb.org/ https://github.com/vpejaver/mutpred2	[112]
LEAP	21	Private	[68]
LYRUS	4	https://github.com/jiaying2508/LYRUS.	[113]

**Table 7:** Advantages and disadvantages of each tool.

Tool	Advantages	Disadvantages
CADD	It supports systematic and objective labeling of variants. It can accommodate almost any feature tied to reference assembly coordinates. It has the capacity to score both coding and non-coding variants.	The label of the training dataset for any given variant provides a low estimate of whether the variant is benign or pathogenic.
PolyPhen-2	It has general robustness.	It has low specificity.
Fathmm-MKL	It can predict coding and non-coding regions.	There are limited non-coding datasets available.
REVEL	It is trained and tested on recently identified diseases and associated variants. It incorporates more individual predictors than prior ensemble methods. The training and testing sets used to train any component predictors have been removed to reduce overfitting.	The reliance on pathogenicity assertions from existing databases and predictors might be inaccurate and incomplete.
CScape	It can predict coding and non-coding regions. It is specifically developed for oncogenic variants.	There is a rareness of validated oncogenic variants in non-coding regions.
DeepDriver	It is cancer specific.	It was only trained on specific cancer types but not all.
DrABC	It is highly specific.	There are few carriers of GPVs in CPGs other than *BRCA1/2*, and their endophenotypes are not well represented.
RENOVO	It relies on fewer features, so it is easier to recollect and apply for features of new variants.	The lack of gene- and disease-specific optimization gives uneven performance across variant classes.
SVFX	The CVD (Cardiovascular disease) cohort in the study had a unique strength of being a careful case–control study.	The lack of high-quality inversions and translocations in public databases limits its applicability to distinguishing disease-associated SVs from benign ones.
Aljarf *et al.*	It tailors gene-specific predictive methods to uncover variant-structure–function relationships.	The number of experimentally validated deleterious variants in *BRCA1* and *BRCA2* is limited. The training data are restricted to defined variants that are in protein regions identified to be involved with impaired DNA repair. The source code is not available.
MutPred and MutPred2	It has better performance over other pathogenicity predictors when information on biochemical, molecular or functional impact is available.	It is based on a small and biased dataset (CAGI dataset).
LEAP	It is usable and interpretable so can combine many different forms of evidence used for expert manual variant classification based on ACMG guidelines.	It needs a more mature database and further model tuning. A disease-specific model needs increased training data size and generalizability. The source code is not available.
LYRUS	Includes the variation number.	Dynamic-based features have a low impact score. It cannot be applied to proteins.

**Table 8:** Overview of the data features used in each tool development.

Tool	Nucleotide-based	Amino acid-based	Protein-based	Conservation-based
CADD	√	√	√	√
PolyPhen-2	√	√	√	√
Fathmm-MKL	√	√	√	√
REVEL	–	√	√	√
CScape	√	√	–	–
DeepDriver	√	–	–	–
DrABC	√	–	–	–
RENOVO	√	–	–	–
SVFX	√	–	–	√
Aljarf *et al.*	√	√	√	√
MutPred and MutPred2	√	√	√	√
LEAP	√	-	√	√
LYRUS	√	√	√	√

**Table 9:** Comparison of the performance of different pathogenicity prediction tools on BC data.

Tools	Relevance			Type (ML/Non-ML)	AUC	Reference
	Trained on conservation data	Trained on BC data	Tested on BC data			
CADD	√	–	√	ML	0.99	[117]
Polyphen	√	–	√	ML	0.88	
Gene-specific model	√	√	√	ML	0.999	
SIFT	√	–	√	Non-ML	0.11	
Polyphen	√	–	√	ML	0.64	[113]
Lyrus	√	√	√	ML	0.89	
SIFT	√	–	√	Non-ML	0.66	
Polyphen	√	–	√	ML	0.628	[71]
CADD	√	–	√	ML	0.621	
Revel	√	–	√	ML	0.63	
SIFT	√	–	√	Non-ML	0.40	
Polyphen	√	–	√	ML	0.77	[101]
SIFT	√	–	√	Non-ML	0.55	
Polyphen	√	–	√	ML	0.87	[118]
Revel	√	–	√	ML	0.97	
SIFT	√	–	√	Non-ML	0.85	

The tools specifically developed and trained on BC data were the most accurate when testing for BC variants, followed by cancer-specific and finally non-disease-specific tools. One of the most accurate tools discussed in this paper is the DrABC tool, as it was developed and trained on BC data. As only a limited number of tools were developed for specific targeted diseases like BC, developing new tools trained on detailed BC data or training existing tools on BC data mostly yields more accurate results for predicting BC pathogenic variants. As proven by several research including Mohannad and Borbala [117], Nikta *et al*. [76] and Hui-Heng *et al*. [72] that when the tool was developed for cancer in specific and was trained on either variants of a specific gene or a collection of variants from different genes, it has shown an enhanced performance compared with the tools developed for general purposes.

## CONCLUSIONS

With the rapid development of genomics and many successful genome projects, the known number of missense variants is increasing rapidly. Thus, it has become essential to learn more about the pathogenicity of such variants to predict, prevent or tailor the treatment for diseases. This review discusses several tools and databases that can be useful in predicting the pathogenicity of variants associated with BC. We provide an in-depth review of diverse databases that can be used, the types of variants included, the accessibility of the underlying data sources and the website of each database. We provide an example of the databases and tools used for prediction of BC pathogenicity. Among all the reviewed databases, we identify that the databases with cancer-specific genetic variants such as NCG, IntOGen and OncoKB are considered strong candidates for training BC-specific pathogenicity prediction tools. The rising issue that states that predisposition gene variants are inherited from humans themselves and not from other primates is not valid on the discussed tools, as none of the tools discussed was trained only on conservation data.

Moreover, a description of each tool, the type of application, the training data, the algorithm realized by the tool and the reliability and accessibility of the source code link were provided. The advantages and disadvantages of each of the discussed tools were provided to aid biomedical researchers in choosing the tool most suitable for a particular research project. The pathogenicity prediction tools DrABC and CScape were shown to have outstanding performance in predicting BC pathogenic variants. We identify that the tools specialized by training on multiple diverse datasets from different databases for the same disease have shown higher accuracy and specificity, thereby helping clinicians in predicting and diagnosing BC as early as possible. The tools discussed in this review are not restricted to BC only; other cancers and sometimes other diseases pathogenic variants can be predicted using the same available tools. The same applies to the databases, in which they are inclusive of variants for several diseases and cancers, not only BC.

**Key Points**

- Review genetic variant databases used specifically for the prediction of breast cancer pathogenicity.
- Review machine learning tools used for breast cancer variants pathogenicity prediction.
- Compare between different genetic variant databases and their influence of the prediction.
- Compare between different machine learning tools and their prediction performance using different genetic variant databases.

## Data Availability

All data generated or analyzed during this study are included in this published article.

## Author Biographies

**Rahaf M. Ahmad **is a dedicated Ph.D. candidate in the Health and Data Science Lab at the United Arab Emirates University-College of Medicine and Health Sciences, she has a Bachelor Degree in Pharmacy, and a Master Degree in Nanoscience and Nanoengineering. With a diverse academic background, she brings a unique perspective to her pioneering research in breast cancer pathogenicity prediction using machine learning. Currently, she is in the 2nd year of her doctoral studies and she has seamlessly integrated her diverse background into her research. Her ability to navigate the interface multidisciplinary fields showcases her commitment to pushing the boundaries of research and innovation. Her academic journey is punctuated by publications, conference presentations and other research events. Her collaborative spirit is evident in her active participation in collaborative projects.

**Bassam R. Ali** is Professor of Molecular and Genetic Medicine and leader of the Genetics and Development Research Priority Group at the College of Medicine and Health Sciences, UAE University. Prof. Ali obtained his PhD degree in biochemistry from the University of Cambridge where he was the recipient of Said Foundation Scholarship and the UK ORS Award. Subsequently, Prof. Ali worked at University College London and Imperial College London before joining the UAEU in August 2006.

**Fatma Al Jasmi** is the Chair of the Genetic & Genomic department at UAE University and a Metabolic consultant at Tawam Hospital. She graduated from UAE University in 2000, pursued postgraduate studies at the University of Toronto, and received the Canadian Board of Pediatrics in 2006. As a founder of the UAE Rare Disease Society, she established the Biochemical Genetic Fellowship and has received numerous awards, including the Prime Minister Award for Excellence (2017) and the Women in Science Hall of Fame recognition (2015).

**Richard O. Sinnott **is the Director of eResearch at the University of Melbourne and Professor of Applied Computing Systems. In these roles he is responsible for all aspects of eResearch (research-oriented IT development) at the University. He has been lead software engineer/architect on an extensive portfolio of national and international projects, with specific focus on those research domains requiring finer-grained access control (security).

**Noura Al Dhaheri **is an Assistant Professor at the Department of Pediatrics, College of Medicine and Health Sciences, certified by the American Board of Pediatrics with qualifications in Medical Genetics & Genomics and Medical Biochemical Genetics. Her current research focuses on Genomic Medicine, particularly in novel gene discoveries for conditions like cleft lip/palate and vertebral malformation. Dr. Al Dhaheri is a part of the Baylor-Hopkins Center for Mendelian Genomics, aiming to elucidate the genetic basis of Mendelian disorders. Additionally, she explores Metabolomics to identify biomarkers for diagnosis and monitoring in patients with metabolic disorders, integrating this with genomics data for better variant annotation.

**Mohd Saberi Mohamad** is a professor of artificial intelligence and health data science. He is now the Director of the Health Data Science Lab in the College of Medicine and Health Sciences, UAE University. His research interests include artificial intelligence, bioinformatics and data science.
